# Editorial: Immune tolerance dual role: advancements in cancer and autoimmune diseases

**DOI:** 10.3389/fimmu.2025.1676002

**Published:** 2025-08-25

**Authors:** Shisan Bao, Xiaoxiao Liu, Chun Jing Wang

**Affiliations:** ^1^ Scientific Research Division, The Third affiliated Hospital, Gansu University of Chinese Medicine, Baiyin, Gansu, China; ^2^ The Affiliated Hospital of Xuzhou Medical University, Xuzhou, China; ^3^ Institute of Immunity & Transplantation, University College London Division of Infection & Immunity, London, United Kingdom

**Keywords:** immune tolerance, cancer, autoimmunity, inflammatory disorder, immune modulation

In the evolving landscape of immunology and oncology, the immune system is increasingly recognised as both a sentinel and a saboteur—capable of maintaining homeostasis or fuelling disease. Advances in immunotherapies and systems-level approaches such as multi-omics have exposed the dual nature of immune regulation: it can suppress tumours or promote their progression, preserve tolerance or incite autoimmunity. This paradox underscores the central challenge in harnessing immunity therapeutically. The studies featured in this Research Topic collectively reflect the complexity of immune modulation and highlight emerging opportunities for context-aware interventions across cancer and inflammatory diseases.

Zeng et al. investigate the PYHIN family—AIM2, IFI16, IFIX, and MNDA—cytosolic DNA sensors that traditionally mediate inflammasome activation and pyroptosis (https://doi.org/10.3389/fimmu.2025.1576674). While these proteins may promote anti-tumour immunity through immune-mediated cell death, they can also enhance tumour progression in certain contexts by sustaining chronic inflammation or aiding immune evasion. Their findings caution against indiscriminate therapeutic targeting and emphasise the need for tumour-specific strategies that consider immune landscape, microenvironment, and PYHIN expression dynamics.

Torniai et al. present a rare case of a patient with sarcomatoid urothelial carcinoma and pre-existing dermatomyositis (DM) who responded to pembrolizumab without autoimmune exacerbation, supported by intravenous immunoglobulin (IVIG) (https://doi.org/10.3389/fimmu.2025.1558964). Although the case suggests a possible role for immunomodulatory co-therapies in managing immune-related adverse events (irAEs), it remains anecdotal. Broader evidence is needed to establish standardised protocols and assess long-term safety of immune checkpoint inhibitors (ICIs) in autoimmune populations.

The review by Huang et al. explores the immunomodulatory potential of mixed-dose radiotherapy (RT) in non-small cell lung cancer (https://doi.org/10.3389/fimmu.2024.1508007). By combining high- and low-dose RT, the authors observed enhanced chemokine expression (e.g. CCL17), increased CD8^+^ T cell infiltration, and improved synergy with ICIs. These findings reinforce RT’s emerging role as an immune-modulating tool, not merely a cytotoxic one. However, optimal fractionation schedules, timing relative to immunotherapies, and tumour-specific responses require further investigation before broad clinical adoption.

In the realm of microbiota and cancer, the review on *Akkermansia muciniphila* highlights its depletion in hepatocellular carcinoma patients with poor ICI response (Yang and Shi). Although mechanistic details remain incomplete, this commensal bacterium appears to influence anti-tumour immunity through gut–liver axis modulation. Translating this into clinical practice will require well-designed trials that carefully standardise interventions and identify optimal microbial strains or metabolites.

Wu et al. report on the traditional herbal formula Yinchenhao decoction (YCHD) in an autoimmune hepatitis mouse model (https://doi.org/10.3389/fimmu.2024.1488125). Their work suggests that YCHD restores Th1/Treg balance and promotes gut-derived short-chain fatty acids, thereby enhancing immune tolerance. While mechanistically insightful, the translation of herbal medicine into clinical immunology remains hindered by challenges in formulation consistency, pharmacokinetics, and reproducibility across batches and populations.

A promising therapeutic innovation is described by Song et al., who evaluate IAH0968—a first-in-class, afucosylated anti-HER2 monoclonal antibody designed to enhance antibody-dependent cellular cytotoxicity (https://doi.org/10.3389/fimmu.2024.1481326). In a heavily pretreated cohort, IAH0968 achieved a disease control rate above 50%, albeit with modest objective responses. This study reinforces the relevance of Fc-engineering in strengthening immune effector functions, though further trials in earlier treatment settings or in combination regimens are needed to realise its full potential.

Chen et al. identify SPAG4 as a novel glioblastoma target associated with disulfidptosis, an emerging form of regulated cell death (https://doi.org/10.3389/fimmu.2024.1462064). Elevated SPAG4 correlated with poor prognosis and increased CD47 expression, a known immune evasion marker. While these findings introduce an intriguing biomarker, future studies must address SPAG4’s tissue specificity, targetability, and functional role within the tumour–immune microenvironment to support its translational utility.

Addressing a diagnostic challenge, Lyu et al. identify serum CCL4 as a candidate biomarker to distinguish gliomas from inflammatory CNS diseases (https://doi.org/10.3389/fimmu.2024.1461450). Their findings suggest that peripheral immune profiling could complement conventional neuroimaging. However, external validation in larger, diverse cohorts is essential before clinical integration.

Finally, Jiao et al. present a sobering case of immune-related haemophagocytic lymphohistiocytosis following tislelizumab treatment in a patient with microsatellite instability-high colorectal cancer and systemic lupus erythematosus (https://doi.org/10.3389/fonc.2025.1585133). Despite rapid intervention, the patient died within 16 days of diagnosis. This rare but fatal irAE illustrates the precarious immunological balance in patients with pre-existing autoimmunity and underscores the urgent need for risk stratification tools and mechanistic insights to guide immunotherapy in high-risk populations.

Taken together, the studies in this Research Topic illuminate the complexity—and, at times, unpredictability—of immune modulation in cancer and inflammatory disorders. They converge on a common message: effective immunotherapy requires nuanced understanding of immune contexture, patient history, and the evolving crosstalk between tumour, host, and environment. Bridging mechanistic discovery with translational insight will be key to transforming immune complexity into therapeutic precision.

